# The Effect of Temperature Increases on an Ant-Hemiptera-Plant Interaction

**DOI:** 10.1371/journal.pone.0155131

**Published:** 2016-07-19

**Authors:** Katayo Sagata, Heloise Gibb

**Affiliations:** 1 Department of Zoology, La Trobe University, Melbourne, VIC 3086, Australia; 2 Papua New Guinea Institute of Biological Research, Goroka, Eastern Highlands Province, Papua New Guinea; Universidade de São Paulo, Faculdade de Filosofia Ciências e Letras de Ribeirão Preto, BRAZIL

## Abstract

Global temperature increases are significantly altering species distributions and the structure of ecological communities. However, the impact of temperature increases on multi- species interactions is poorly understood. We used an ant-Hemiptera-plant interaction to examine the potential outcomes of predicted temperature increases for each partner and for the availability of honeydew, a keystone resource in many forest ecosystems. We re-created this interaction in growth cabinets using predicted mean summer temperatures for Melbourne, Australia, for the years 2011 (23°C), 2050 (25°C) and 2100 (29°C), respectively, under an unmitigated greenhouse gas emission scenario. Plant growth and ant foraging activities increased, while scale insect growth, abundance and size, honeydew standing crop per tree and harvesting by ants decreased at 29°C, relative to lower temperatures (23 and 25°C). This led to decreased scale insect infestations of plants and reduced honeydew standing crop per tree at the highest temperature. At all temperatures, honeydew standing crop was lower when ants harvested the honeydew from scale insects, but the impact of ant harvesting was particularly significant at 29°C, where combined effects of temperature and ants reduced honeydew standing crop to below detectable levels. Although temperature increases in the next 35 years will have limited effects on this system, by the end of this century, warmer temperatures may cause the availability of honeydew to decline. Decline of honeydew may have far-reaching trophic effects on honeydew and ant-mediated interactions. However, field-based studies that consider the full complexity of ecosystems may be required to elucidate these impacts.

## Introduction

Global surface temperature has increased by approximately 0.6°C in the past century [1] and this change is beginning to have a significant impacts on biological systems [2]. Specific mechanisms through which climate change, particularly temperature, affects species are complex. Temperature may influence species by directly altering population densities, survival and fecundity [3–5] or indirectly through its effect on interacting species, food sources, natural enemies, competitors and species traits [6–9]. Insects are likely to respond quickly to increasing ambient temperature as they are ectotherms [3, 10]. For sap-sucking hemipterans (e.g., aphids, scale insects, membracids and mealbugs), temperature commonly has strong direct effects on population dynamics [11]. For example, an increase in sap-sucking insect densities is likely to result in high plant infestation with cascading trophic effects.

Climate change may also indirectly influence sap-sucking insects through effects on their host plant physiology and chemistry. Elevated CO_2_ and temperature increase the C:N ratios of plants [12]. To compensate for nitrogen imbalance, insect herbivores increase consumption [13]. For sap-sucking insects, this means a high honeydew (a sugar-rich substance) excretion rate which then supports a third trophic group of consumers, which are commonly mutualists. Only a few studies have investigated the cascading effects of climate change on this common tri-trophic interaction [9, 14–16]. As with other interactions [14], warmer temperature and changes in other climate variables may impact differently upon different partners. Traits of each partner such as growth, reproduction, and foraging patterns may respond differently and simultaneously, making empirical investigations more challenging. However, insights into how complex interactions respond to warming environments are critical to understanding how real-world communities will respond to a changing climate.

Many organisms use honeydew as food [17–20], but ants are by far the most copious consumers of honeydew [21]. Ant associations with honeydew producing sap-sucking insects are well known. Honeydew acts as a keystone resource in many forest systems [19, 20, 22], promotes ant-driven ecosystem processes such as seed dispersal and predation [23] and alters ant communities [24, 25]. Dominant ants forming mutualistic relationships with sap-sucking insects can have significant impacts on local biodiversity [26] and complicate pest management practices in agricultural systems [27]. Any changes in interactions between ants and their honeydew providers may thus have broad impacts on ecosystems [21].

The importance of ant-Hemiptera mutualisms likely depends on the availability of honeydew and other sources of liquid carbohydrates (floral, extrafloral nectar). Sap-sucking insects can produce significant amounts of honeydew [19] and ants can harvest over 50% of this energy [20]. However, environmental factors such as temperature affect population dynamics of sap-sucking insects [28, 29], which directly affects honeydew production [30, 31]. Yet, the impact of increasing temperature on sap-sucking insects and honeydew production is poorly understood, though it is expected that insect herbivory will increase in response to temperature increases [11, 32]. An increase in the feeding rate of sap-sucking insects may result from high population density or a low level of Nitrogen (N) in the sap. More honeydew may become available during periods of increasing temperature. However, if temperature continues to increase, sap-sucking insects may not survive and honeydew production may decline. Under an unmitigated greenhouse gas emission scenario, annual mean maximum temperature in Australia over the next century is expected to increase by 5–6°C [33]. Understanding how each partner in the ant-sap-sucking insects-plant interaction responds to changing temperature is critical to maintaining the many important ecological interactions mediated by honeydew and ants.

Here, we test how increasing temperature affects an ant-scale insect -plant interaction. We used the interaction between the sap-sucking scale insect *Eriococcus coriaceus* its host plant *Eucalyptus camaldulensis* and the native ant *Iridomyrmex rufoniger* as a model system to test the effect of increasing temperature. We predicted that temperature increases will have positive effects on: (i) growth and biomass of *E*. *camaldulensis*; (ii) *E*. *coriaceus* population dynamics; (iii) honeydew standing crop per scale insect and tree; (iv) ant harvesting of honeydew; and (v) ant activity.

## Materials and Methods

### Study system

Temperature in Australia is predicted to increase significantly over the next 100 years under a range of different greenhouse gas emission levels and mitigation scenarios [33]. Here, we used the high greenhouse gas emission and no mitigation scenario (A1F1). Under this scenario, temperatures in Australia are predicted to increase approximately 3 and 6°C for the years 2050, 2100, respectively. We added these temperature increases to the 2011 mean maximum (24°C) for spring and summer (September-February) for Victoria (http://www.bom.gov.au/climate/data/), to which the study species (described below) are native. In 2011, 2050, and 2100, the mean maximum expected temperatures for spring to summer in Victoria were predicted to be 24, 27 and 30°C respectively (Table 1).

**Table 1:** Maximum (day) and minimum (night) actual temperatures and growth cabinet size for the three temperature treatments, representing current temperatures and predicted temperatures for 2050 and 2100. Numbers in brackets are the temperatures achieved in the growth cabinets ± standard deviations.

**Temperature (°C)**	**2011**	**2050**	**2100**
Increment	0	3	6
Maximum	24 (23) ± 0.56	27 (25) ± 0.67	30 (29) ± 0.48
Minimum	12 ± 0.48	15 ± 0.28	18 ± 0.43
Cabinet size (l x w x h-cm)	186 x 74 x 114	130 x 120 x 156	190 x 76 x 152

River red gum (*E*. *camaldulensis)* and sap-sucking scale insect (*E*. *coriaceus*) were selected as the model system based on their host relationship, geographical distribution and easy access to sampling honeydew. The river red gum is native to Australia and is widely distributed (http://chah.gov.au/avh/public_query.jsp). The scale insect is found mostly in cooler regions of Australia where annual mean maximum temperature ranges from 12–24°C, although it has been reported from the tablelands in Queensland, where the annual mean maximum temperature is 27°C [34]. Field populations were collected from La Trobe University campus and from La Trobe University Wildlife Sanctuary. *Eucalyptus camaldulensis* seedlings were bought from Wimmera Native Nursery (36°26'16.21"S, 142° 1'2.69"E) and grown for 3.5 months in 15 cm diameter plastic pots with native potting mix. During the first two months plants were infested with *E*. *coriaceus* by tying infested twigs and leaves onto the stems. Water was provided via the trays in which the plants were grown. Liquid fertilizer was applied to the plants during re-potting and two weeks before placing them in the growth cabinets (see experimental design).

*Iridomyrmex rufoniger* is native ant common throughout Australia, with colonies containing thousands of workers in interconnected nests [35]. Field colonies of *I*. *rufoniger* were collected from La Trobe University Wildlife Sanctuary using a battery-driven hand held vacuum cleaner and an aspirator. All nests were within an area of about 0.5 ha and were separated by approximately 20 to 160 m.

The ants were kept in the laboratory for 2.5 months prior to using them in the experiment. These colonies were kept in plastic containers (15 x 10 x 7 cm, l x w x h), with three to five 10 ml plastic centrifuge tubes as nest sites, each of which was one-third filled with water and stopped with cotton balls. The ants were incubated in a growth cabinet at 24°C (day) and 10°C (night) temperatures and a 12:12 hour light- dark cycle and were fed 25% sugar water and 2–3 freshly squashed crickets (*Teleogryllus oceanicus*) three times per week. Colonies from different nests did not interact aggressively with each other, which suggests that we sampled from a large, polydomous colony [35] or a supercolony.

### Experimental design

To simulate expected temperature increases, this study was conducted in three growth cabinets varying in size, with each cabinet set to specified maximum (day) and minimum (night) temperatures (Table 1). Each cabinet was divided into treatments with ants and without ants to test the effects of ants and temperature on honeydew standing crop. Each ant treatment had eight potted *E*. *camaldulensis* placed in a tray, with one plant per pot (15 cm diameter). All plants had similar height across three temperature treatments (ANOVA; *F*_2, 45_ = 1.891, *P* = 0.163) which ranged from 100–190 cm and had 1–182 *E*. *coriaceus* adults and instars. Temperature inside the growth cabinets was monitored with ibuttons set to record every 20 minutes. The experiment was run for 62 days.

Ant treatment consisted of five *I*. *rufoniger* colonies per growth cabinet, with each colony consisting of 500 workers. However, there was shortage of queens so only three colonies had a queen each while the other two colonies had 200 brood each initially and were supplemented with 200 brood twice during the course of experiment to maintain similar brood levels amongst colonies. Placing brood in queenless colonies may allow workers to perform normal colony functions [36]. The colonies were placed in smaller plastic containers of the same size and treatment described earlier, except that they had up to six 1 mm exit holes on one of the sides. The holes were large enough to allow access of workers but not the queens. These small containers were placed inside a larger plastic container (18 x 11.5 x 8.5 cm, l x w x h) and placed next to the growth trays. Ant access to trees was facilitated by connecting the larger plastic containers with plastic tubing (4.9 mm diameter). One end of the tube was tied to the plants ~ 3–5 cm from the pot surface. Wooden spatulas were used to bridge the pots so that all the five colonies had access to all the plants as different colonies were observed not to interact aggressively with one another.

A cardboard barrier with holes that fitted securely around each pot was fitted to each tray to prevent ants from drowning in the water. Then the sides of the trays were sealed with duct tape and greased with tanglefoot to prevent ants from escaping. A small plastic container with the bottom removed was fitted to one of corners of the tray for watering. Four freshly squashed crickets and 4–6 g of scrambled egg were provided twice per week as protein source for the ants.

### Observations

#### *Eucalyptus* growth and primary productivity

Effects of temperature and scale insects on the growth and primary productivity of *E*. *camaldulensis* were examined by measuring plant height and biomass. Height was determined by measuring the growth of main stems and branches. Regions close to the meristem were marked with a permanent marker and the height to the meristem was measured and counted before and after the experiment. The difference in height indicated growth of the trees. The average height gained across a single plant was used as the response variable in the analysis. The plants were harvested at the end of the experiment and oven dried at 55–60°C for 48 hours to determine their biomass. The roots were thoroughly washed with water and pre-dried at 30°C for 30–60 minutes to remove excess moisture before including them with rest of the plant material. It is difficult to measure plant biomass before the experiment. However, gain in biomass is related to growth in many *Eucalyptus* species [37]. Therefore, we assumed that height gained provides an indication of biomass gained at the end of the experiment.

#### Scale insect population dynamics

Per capita growth rate, per capita instar production and size (head body length of adults) of the scale insect were used as measures of population dynamics. The effect of temperature on the growth rate of scale insects was examined by measuring per capita instar production and per capita growth rate. Per capita instar production was the final number of instars divided by number of adult scale insects at the end of the experiment. Per capita growth rate was the final number of adult scale insects divided by the initial number of adult scale insects. Instars and adult scale insects were easily distinguished as felted sacs are present only in adults [28]. The effect of temperature on scale insect size was determined by measuring body length (tip of the head to end of anus ventrally) of the adults. The number of individuals used to obtain mean measures varied from 2 to 5 individuals, depending on the number of scale insects available per tree. The measurements were taken at the end of the experiment to avoid altering feeding activity and reproduction. We used a stereo microscope (Leica^®^ M165C, Switzerland) with camera attached to take live measurements using Leica Application Suite (LAS) imaging software (Version 3.4.0). Felt sacs were removed before taking the measurements.

#### Honeydew standing crop

To test for the effect of temperature and ants on honeydew standing crop per tree and scale insects, we sampled honeydew present on the anus of the scale insects using filter papers [38]. Honeydew standing crop is the mass of honeydew available at any given point in time which can be affected by consumers and the environment [22, 31]. Honeydew was removed from all scale insects one hour prior to sampling to allow us to determine the mass of honeydew available on a tree per hour. Honeydew was sampled at 15 minute intervals for 60 minutes per growth cabinet once or twice per week for eight weeks. Honeydew on the anus of the scale insects was absorbed onto small pieces of cut filter papers that had been pre-weighed and oven dried at ~ 45°C for 24 hours. Each piece of filter paper was kept separate to avoid fluids being exchanged and care was taken to reduce smearing on storage containers. Filter papers were again oven dried at ~ 45°C for 24 hours and re-weighed using microbalance (Mettler Toledo^®^ XS3DU, Switzerland, accurate to 0.0001 mg). The difference in the mass of the filter paper provided a measure of honeydew (dry mass) available per hour. Honeydew standing crop per insect was determined by dividing honeydew dry mass per plant by the total number of scale insects on that plant.

#### Honeydew harvesting and ant activity

To test for the effect of temperature on the mass of honeydew harvested, we weighed six ants (per cabinet) observed tending the scale insects or feeding on honeydew that had distended gasters and another six ants exiting the nest or moving around on the surface of the plant pots without distended gasters using a Mettler Toledo^®^ microbalance. The difference in mean weights between these groups provided a measure of the honeydew harvested by an average worker. The ants were aspirated into a small pre-weighed plastic vial with the top half of the vial painted with fluon (to prevent ants escaping) and the weight of the pre-weighed vial was subtracted from the weight of the vial with ants to determine the weight of the ants. Aspirating the ants may have provoked them to release defensive or alarm chemicals, potentially reducing their weight. However, we have no reason to expect differences in this response between ants with empty or distended gasters. All ants were kept in vials to avoid re-sampling until sampling was completed and then returned to their cabinets. Observations were made within 30–60 minutes per cabinet once or twice weekly for eight weeks. However, in the cabinets at 23 and 25°C, less than six or no ants were observed harvesting honeydew on any one tree, which limited the number of ants sampled per tree. Therefore ants were sampled across the trees rather than per tree in all the cabinets.

The effect of temperature on ant activity was measured by counting the number of ants ascending/descending trees per minute for 60 minutes per cabinet once per week for five weeks. The number of ants was counted across all trees because in growth cabinet 29°C there were many ants (78–238) that moved very fast and took more than one minute to count ants per tree. This made it difficult for one observer to count ants on individual trees. Observations were made by one person spending an hour at each cabinet each sampling period.

This study was conducted with permission from Department of Environment and Sustainability, Victoria under license number 10005518 and with permission from La Trobe University Wildlife Sanctuary. For *E*. *camaldulensis* no specific permissions are required as it is widely sold in native nurseries, while *I*. *rufoniger* is not endangered or protected species.

### Statistical analysis

A log-linear model based on the Poisson link function was used to test for the effect of temperature, scale insect abundance and ants on the growth of *Eucalyptus* followed by Tukey contrast test. Scale insect abundance was included as another predictor in the model because scale infestation can reduce the growth of *Eucalyptus* [39]. *Eucalyptus camaldulensis* biomass and *E*. *coriaceus* growth rate and brood production data were analysed with two-way ANOVA, followed by post-hoc Tukey tests. Honeydew standing crop data were analysed with linear mixed-effects regression (LMER) based on a REML (residual maximum likelihood estimation) approach. Some trees had no scale insects thus no honeydew. Such unbalanced data with repeated measures are appropriately analysed with REML than traditional ordinary least square methods [40, 41]. Ant attendance was used as another predictor because attending ants can have positive effects on the survival of sap-sucking insects [42] and honeydew production [43]. Data were log, square root or fourth root-transformed to meet the assumptions of normality and homogeneity of variances for ANOVA tests. All analyses were conducted using R 2.15.2 statistical software [44]. Linear mixed- effects regression was implemented with *lme* function in *nlme* package. Ant harvesting of honeydew and activity data were not analysed statistically because observations were not replicated across experimental units (i.e. plants). Daily mean temperature in each growth cabinet was slightly lower than the predicted temperatures (see S1 Predicted Temperatures). Therefore, all the analyses were based on actual temperatures in the growth cabinets rather than the predicted temperatures.

## Results

### *Eucalyptus* and scale insect performance

**Fig 1:**
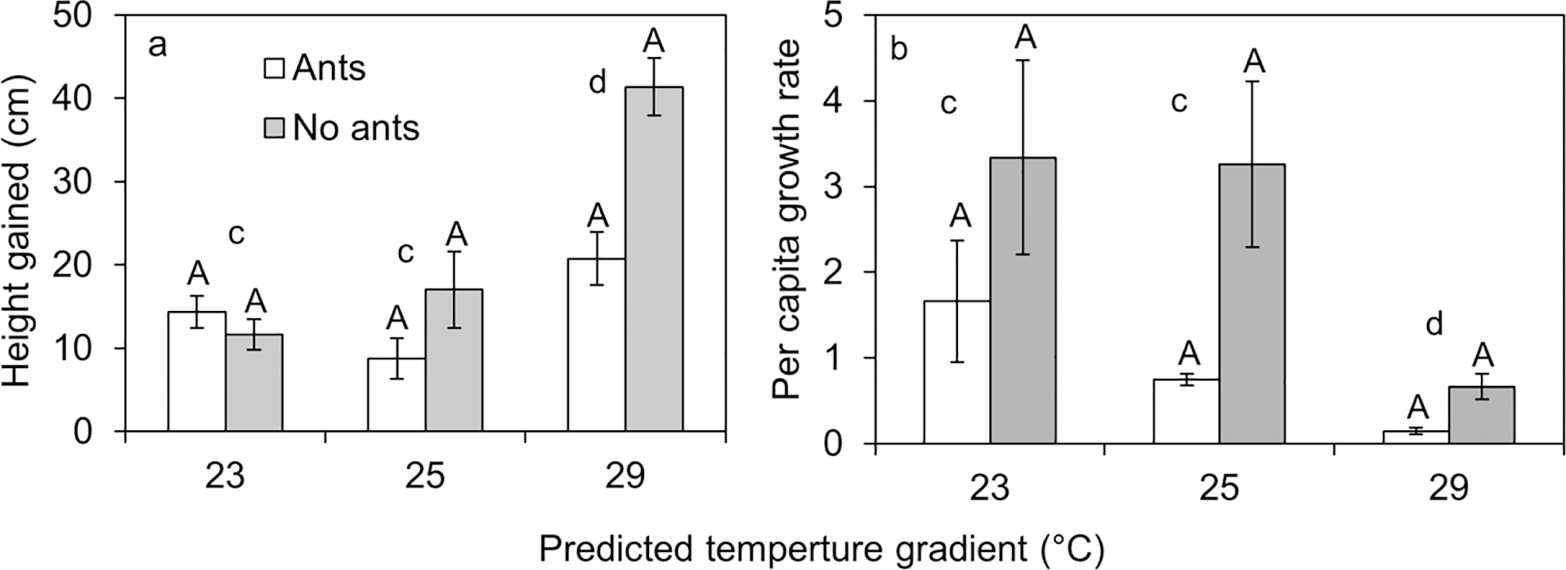
**Mean ± SE *Eucalyptus camaldulensi*s growth (a) and mean per capita growth rate of *Eriococcus coriaceus* (b).** Different upper case letters (between ant treatments) and small letters (between temperature regimes) above the bars show significant differences according to Tukey contrast or post hoc Tukey tests (*P*<0.001). Per capita instar production for *E*. *coriaceus* show similar trends as per capita growth rate. Temperature gradient 23, 25 and 29°C represents predicted temperatures for the years 2011, 2050 and 2100 respectively.

**Fig 2:**
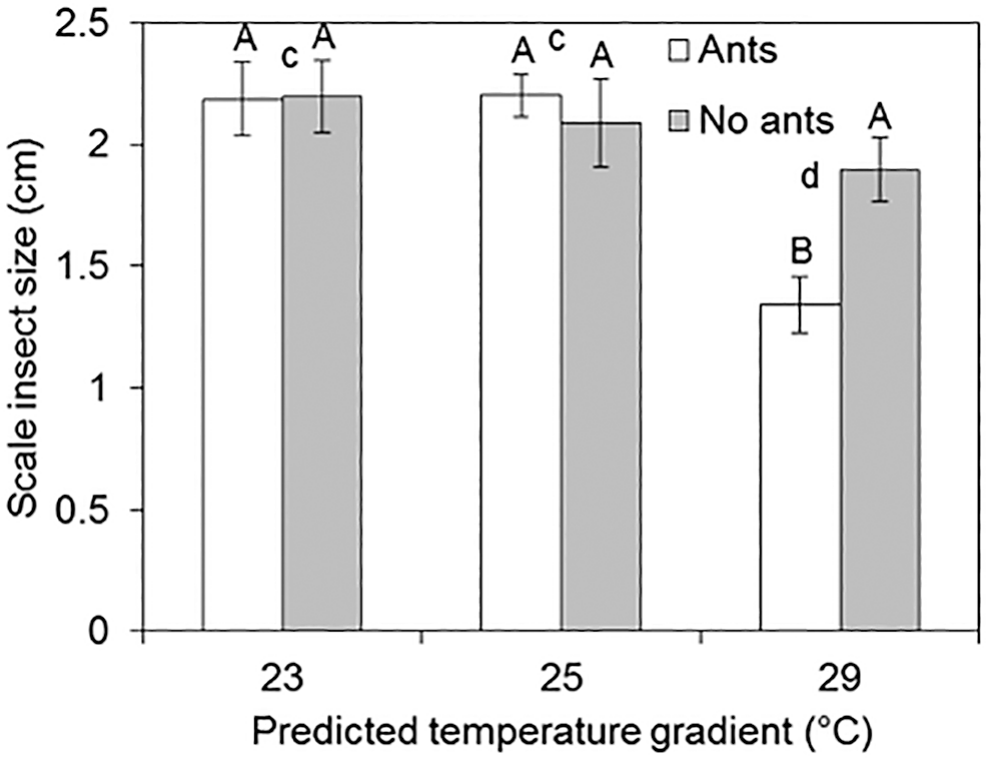
Mean ± SE *Eriococcus coriaceus* size. Different upper case letters (between ant treatments) and small letters (between temperature regimes) above the bars show significant differences according to post-hoc Tukey tests (*P*<0.001). Temperature gradient 23, 25 and 29°C represents predicted temperatures for the years 2011, 2050 and 2100 respectively.

Temperature had a significant effect on the growth of trees but not scale insects, ants or their interactions (Table 2A). The significant effect of temperature on trees was mainly due to trees gaining more height at 29°C than 23 and 25°C according to Tukey contrast test (Fig 1A). The mean dry weight of trees at 29°C was slightly higher (21.48 ± 2.26) than 23 (19.09 ± 1.26) or 25°C (17.39 ± 1.02) but these differences were not statistically significant (Table 2A). Temperature again had a significant effect on scale insect growth rate, instar production (Table 2B). A post-hoc Tukey test showed that these effects were due to a low growth rate at 29°C compared with 23 and 25°C Fig 1B). There was no effect of ants or combine effect of ants and temperature on scale insect growth rate and instar production. However, interaction between ants and temperature had a significant effect on the size of scale insects (Table 2B). Scale insects with ants at 29°C were significantly smaller than those with no ants and those in both ant treatments at 23 or 25°C according to post-hoc Tukey test (Fig 2). In general, all the indicators of scale insect population growth such as per capita growth rate (Fig 1B), showed reduced performance with increasing temperature.

**Table 2:** The results of log linear model and ANOVA on *Eucalyptus* growth and dry weight respectively (a) and ANOVA on scale insect growth and size (b). Significant values are in bold.

**(a). *E*. *camaldulensis***										
**Predictor variable**	*Growth*				*Dry weight*					
	***DF***	***χ***^***2***^	***P***	***DF***	***MS***	***F***	***P***			
Temperature	2	10.911	**<0.001**	2,42	0.021	1.187	0.317			
Scale insect	1	0.016	0.900	1,42	0.017	0.937	0.339			
Ant	1	2.655	0.103	1,42	0.029	1.626	0.210			
Temperature x scale insect	2	0.927	0.628	2,42	0.007	0.392	0.679			
Temperature x ant	2	1.563	0.457	2,42	0.034	1.922	0.161			
Scale insect x ant	1	0.010	0.920	1,42	0.000	0.019	0.892			
Temperature x scale insect x ant	2	0.406	0.816	2,42	0.007	0.357	0.702			
**(b). *E*. *coriaceus***										
		*Per capita growth rate*			*Per instar production*			*Scale insect size*		
**Predictor variable**	***DF***	***MS***	***F***	***P***	***MS***	***F***	***P***	***MS***	***F***	***P***
Temperature	2,45	0.818	13.000	**<0.001**	6.058	23.136	**<0.001**	1.614	10.599	**<0.001**
Ant	1,42	0.002	0.031	0.860	0.026	0.099	0.755	0.271	1.781	0.189
Temperature x ant	2,42	1.128	2.865	0.068	0.591	1.128	0.333	0.509	3.449	**<0.050**

### Honeydew standing crop

**Fig 3:**
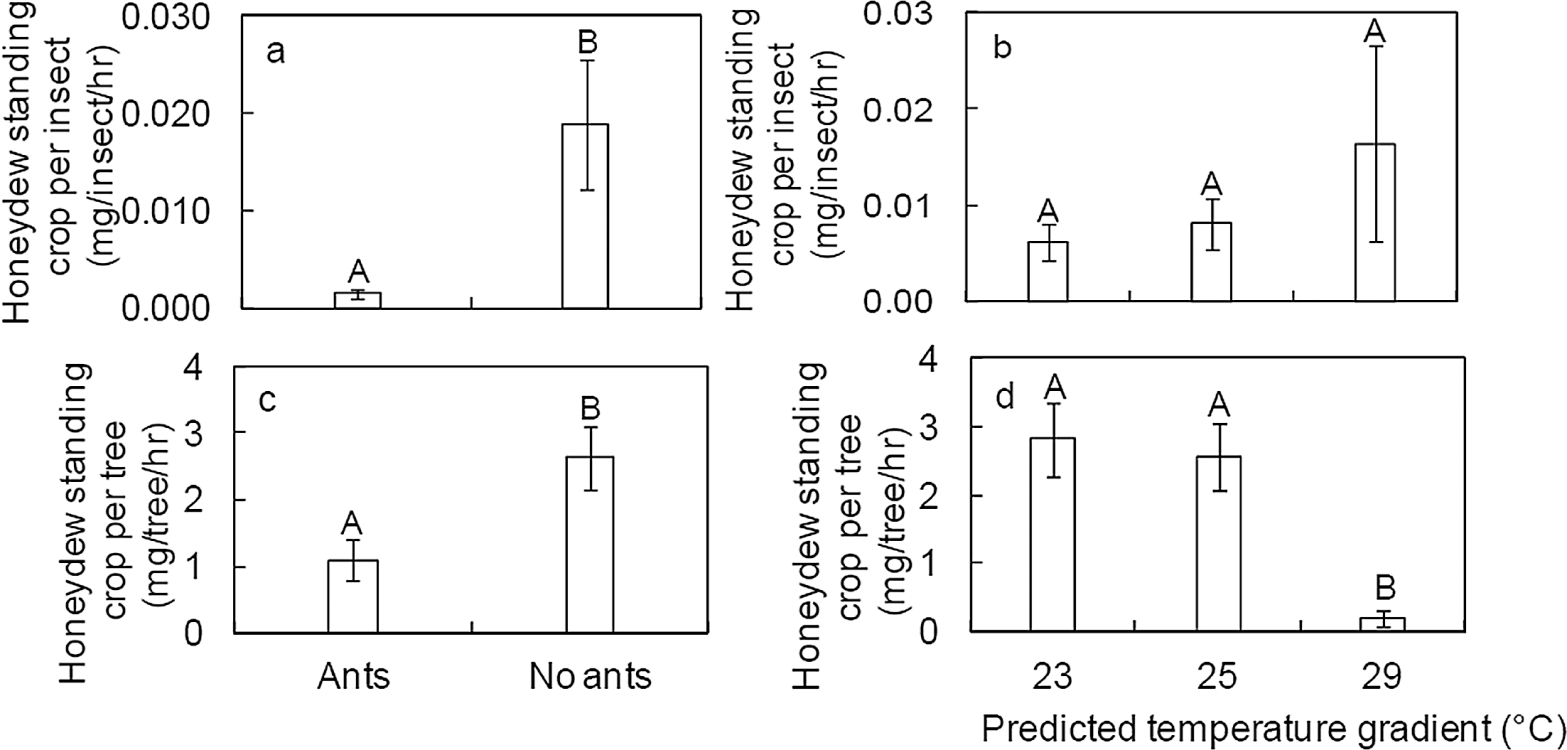
**Effect of ants (a) and temperature (b) on Mean ± SE honeydew standing crop per scale insect and effect of ants (c) and temperature (d) on honeydew standing crop per tree.** Different upper case letters above the bars indicate significant differences according to linear mixed- effects regression (*P* <0.05).

Honeydew standing crop per insect was significantly affected by ants (*df* = 42, *t =* -2.570, *P =* 0.012, Fig 3A), but not temperature (*df* = 2, *t* = 0.166, *P* = 0.884, Fig 3B) or their interaction (*df = 40*, *t* = -1.444, *P* = 0.156). Ants removed most of the honeydew available per scale insect in comparison to treatments with no ants (Fig 3A). However, there was a significant main effect of both ants and temperature on honeydew standing crop per tree (ants, *df* = 42, *t* = -3.328, *P* = 0.002; temperature, 42, *t* = -4. 868, *P<*0.001). Again, there was no significant interaction effect (*df* = 42, *t* = 0.770, *P* = 0.445). That is, less honeydew was present in the treatment with ants than in the treatment with no ants (Fig 3C), indicating that ants were removing honeydew. Along the temperature gradient, honeydew standing crop per tree at 29°C differed significantly (*df* = 42, *t* = -4. 868, *P*<0.001) from both 23°C and 25°C, but there was no difference between 23 and 25°C (*df* = 42, *t* = -0.891, *P* = 0.378). The lower honeydew crop per tree at 29°C is the result of negative effect of elevated temperature on size and number of scale insects per tree (Table 2B).

### Honeydew harvesting and ant activity

**Fig 4:**
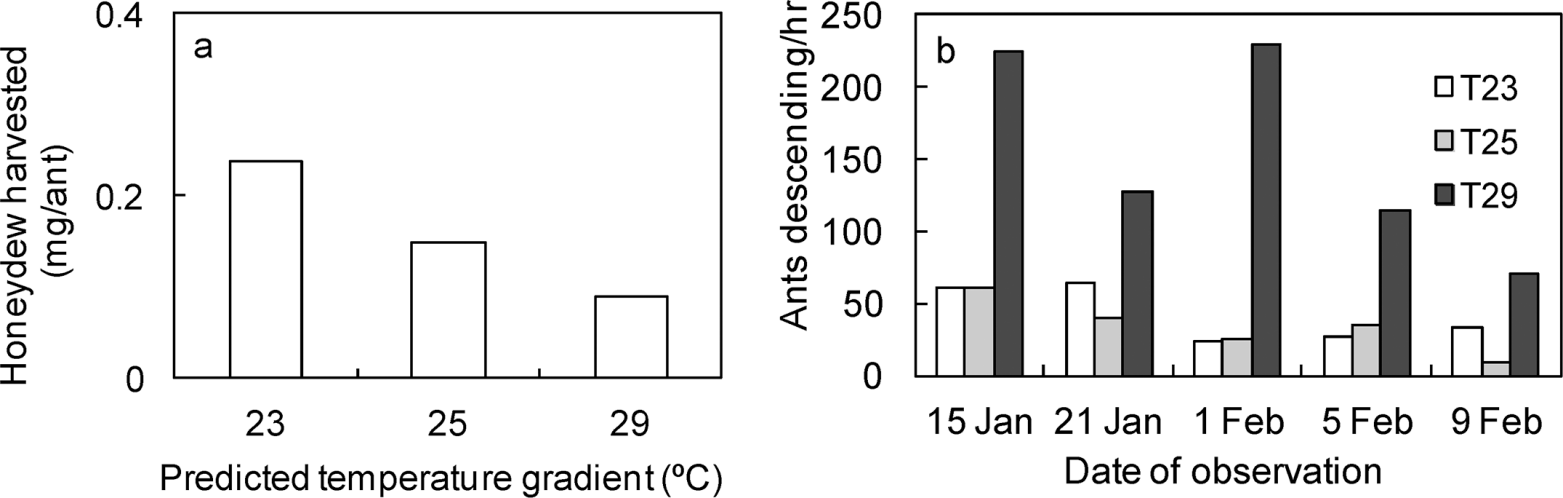
Mean honeydew harvested per ant (a) and ant activity (b) under three experimental temperature regimes.

Ants harvesting honeydew at each temperature weighed more than ants not harvesting honeydew (Wilcoxon signed rank test, V = 21, *df* = 4, *P* = 0.031). Thus, on average, the weight of loads carried by individual ants decreased with increasing temperature (23°C = 0.24 mg; 25°C = 0.15 mg; 29°C = 0.09 mg) (Fig 4A). We also observed higher ant activity (ascending/descending) at 29°C than 23 and 25°C (Fig 4B).

## Discussion

**Fig 5:**
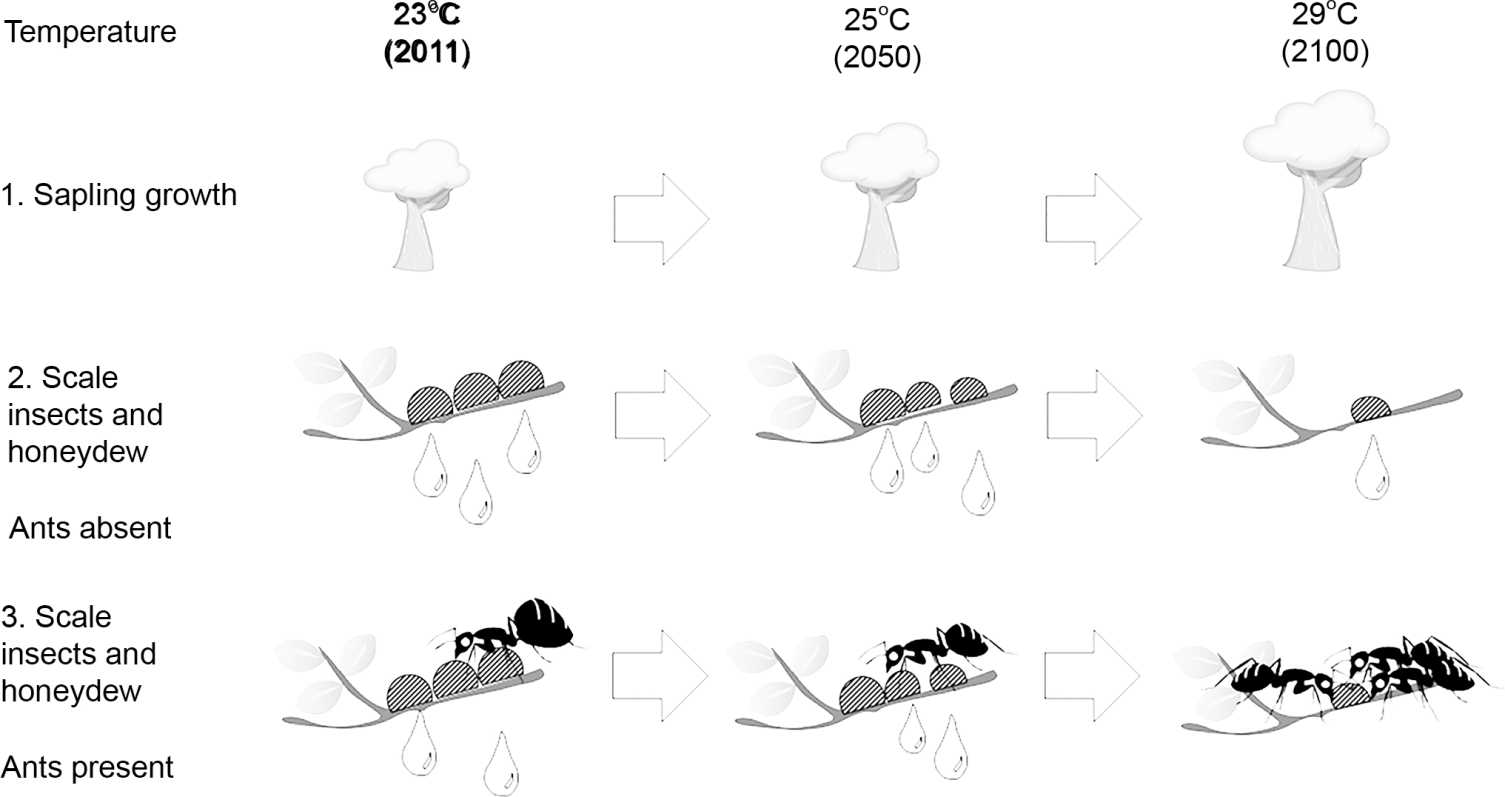
Observed response of each partner in the ant-scale insect-plant interaction to temperature. The biomass of *E*. *camaldulensis* increased with increasing temperature, but at high temperatures (29°C), honeydew production by scale insects declines. Ants decrease the standing crop of honeydew, but honeydew availability is negligible at high temperatures when ants are present.

We manipulated one climate variable: temperature, and observed responses of each partner in an ant-scale insect-plant interaction. The results, summarized in Fig 5, show strong effect of increasing temperature on the growth of plants and scale insects, honeydew standing crop per tree and harvesting by ants but not on honeydew standing crop per scale insect. These findings allow us to predict how each partner in the ant-scale insect-plant interaction may respond to increasing temperature. We also discuss how increasing temperature may affect honeydew production and availability and flow-on effects on ants that primarily feed on liquid carbohydrates.

### *Eucalyptus* and scale insect performance

Elevated temperature (29°C) positively influenced the growth of *E*. *camaldulensis*, which is consistent with findings of other studies [45, 46]. The high growth of *E*. *camaldulensis* at elevated temperature is not surprising because this species thrives in many habitats and temperature ranges. *E*. *camaldulensis* is thus likely to be adapted to cope with a broad range of temperatures. Such wide ranging species provide a readily available host for sap-suckers such as *E*. *coriaceus*. However, when temperatures are warmer during spring and summer, an increase in *E*. *coriaceus* infestation levels can affect the growth of plants [38]. Although we found no significant effect of scale insect infestation, we observed one plant heavily infested with scale insects dying at 25°C, suggesting negative impacts of infestation. However, lethal effects of elevated temperature on *E*. *coriaceus* annulled the negative impacts of scale insects on *E*. *camaldulensis* growth at 29°C, which on average had higher growth (31 cm) than at 23°C (13 cm) or 25°C (13 cm). An increase in growth with temperature should result in more C being stored as biomass [45], while scale insect infestations would reduce biomass. This means that plants growing at 29°C should have gained more biomass than plants growing at 23 and 25°C. However, the increase in biomass gain at 29°C was not significant, possibly because this temperature is close to the physiological limits of *E*. *camaldulensis* [47, 48]. Biomass in leaves, stems and buds could be lost through the scale insects as these are the parts of the plant where sap-sucking insects commonly feed [38, 45]. However, low scale insect density at elevated temperatures means biomass loss through herbivory by scale insects is probably not significant.

Among many factors that interact to regulate the population dynamics of sap-sucking insects, seasonal temperature variation is a key factor. *Erioccocus coriaceus* density is highest when the temperature is lower (late winter and spring) [29]. The low abundance of *E*. *coriaceus* during summer is linked to low fecundity of females and high predation rates [29]. We found that elevated temperature led to reduced population density of *E*. *coriaceus* and slowed its growth rate (Fig 1B) while combine effect of temperature and ants reduced *E*. *coriaceus* body size. The body size-fecundity relationship was observed in earlier studies, where larger female *E*. *coriaceus* produced more instars than smaller females [28, 29]. Small size and low growth rate at elevated temperature indicates poor nutrition or stressful physiological conditions. Ants can encourage sap-sucking insects to increase honeydew production [43, 49]. With less honeydew available for the ants at 29°C, ants are likely to encourage *E*. *coriaceus* to produce honeydew more frequently than they should. Although not significant this, high honeydew availability per scale insect at 29°C compared to 23 and 25 suggest influence of ants on honeydew production (Fig 3B). If ants are encouraging *E*. *coriaceus* to produce honeydew more frequently, *E*. *coriaceus* may be able to afford less time to assimilate nutrients from the sap. Besides, climate warming is associated with higher CO_2_, which is likely to affect nutritional value of the sap [47, 48]. However, we did not measure CO_2_ in this study and the combined effects of higher temperature and CO_2_ on scale insects and honeydew production may differ from their individual effects. Instead, we found that elevated temperature increased plant biomass (Fig 1A), which we would expect to increase scale insect feeding rates, resulting in more honeydew being produced at 29°C. However, this was not the case (Fig 3), suggesting that scale insects did not feed well at elevated temperatures. Elevated temperature beyond the optimum range negatively affects insect physiological functions [50–53]. Therefore, at 29°C, temperatures may have been beyond the limits for the scale insects to function normally, directly affecting their size, growth and feeding behavior.

### Honeydew standing crop, harvesting and ant activity

We expected temperature and the presence of ants to increase honeydew standing crop per insect. However, we found no effect of temperature, which can be partly explained by the feeding behavior of the scale insects. In the aphid *Tuberolachnus salignus*, honeydew excretion at 20°C was close to rate at which sap was forced through the stylets by the turgor pressure of the plants [54]. Scale insects are therefore likely to be feeding at their maximum rate at 23°C, such that increasing temperatures to 25 and 29°C does not allow greater ingestion of sap by scale insects. When we considered the total honeydew standing crop per plant, we found negative effects of elevated temperatures (Fig 3C). This was expected, since scale insect density and growth were higher at lower temperatures so the mass of honeydew available was directly related to scale insect density. It is likely that high instar production and population density at lower temperatures resulted in greater honeydew production than elevated temperature. Favourable temperatures may have also increased the flow of phloem sap by reducing sap viscosity or by increasing the turgor pressure of plants [48]. Since ants attending sap-sucking insects can enhance honeydew production by rapidly stroking the posterior end of the sap-sucking insects with their antennae [43, 49], honeydew production might have been higher in treatments with ants. However, we did not measure this, so can only comment on the observed standing crop per tree.

Our data suggest a strong effect of elevated temperature on the mass of honeydew harvested by individual ants, but a lack of independent replication for this component of the study limits interpretation. At elevated temperatures, honeydew availability was low, so less honeydew was harvested per ant than at lower temperatures (Fig 4A). Low honeydew availability at 29°C meant individual ants carried very low loads on average (0.09 mg), compared with ants at lower temperatures (23°C = 0.25 mg, 25°C = 0.15 mg). When resources are scarce, colonies must somehow maintain their energy intake. This might have been achieved by colonies increasing their foraging activities by recruiting more foragers to the resource. We observed high ant activity (ascending/descending) per minute at 29°C, suggesting that ants were removing most of the honeydew. However, at lower temperatures, where honeydew availability was high and ant activity was low, individual ants carried greater loads on average than ants at 29°C. It seems that ants were behaving in such a way to maximize their energy intake per unit time [55]. We suggest that it may have been less energetically expensive for ants at elevated temperature to forage.

Although honeydew availability depended on ant presence and temperature, it is possible that honeydew quality may also have been affected. High temperature [56], ants and host plants [57] can alter the nutritional content of honeydew. Changes in quantity and quality of honeydew may also affect ant colony nutritional requirements and ant preference for sap-sucking insects [58]. Honeydew quantity and quality may play an important role in defining ant community organization with dominant ant species dominating nutritious honeydew at the expense of less competitive species [25, 59].

If climate change in Australia follows current predictions, our study suggests that *E*. *coriaceus* will do well and honeydew availability will remain high between 23 and 25°C (Fig 5). However, before or by end of this century (2100), we predict that the warmer climate (≥ 29°C) may lower the availability of honeydew, affecting liquid feeding ants such as *Iridomyrmex* and their interactions. However, we focussed on a scale insect species that is limited to cooler regions and was negatively affected by increasing temperature. The loss or decline of sap-sucking insects in cooler regions may reduce the amount of honeydew available to tending ants, unless these losses are supplemented by other species better adapted to warmer climates. Field-based studies that consider the full complexity of ecosystems may be required to elucidate these potential impacts.

## Supporting Information

S1 Predicted Temperatures Temperature fluctuation in the growth cabinets for the years 2011 (a), 2050 (b) and 2100 (c). SE is standard error.
(TIF)
