# Supplementary figures and images for: The Effect of Temperature Increases on an Ant-Hemiptera-Plant Interaction
Source: PLoS One. 2016 Jul 19;11(7):e0155131. doi: 10.1371/journal.pone.0155131 (PMC4951116; doi:10.1371/journal.pone.0155131)

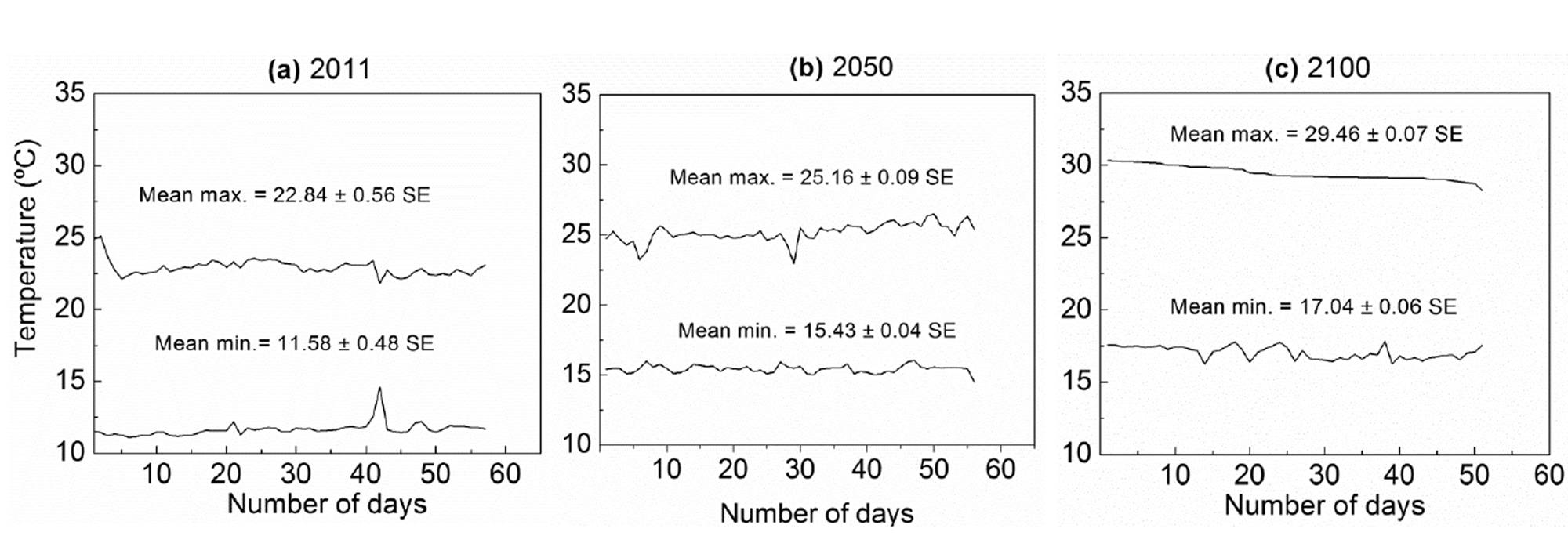

Supplement: S1 Predicted Temperatures — Temperature fluctuation in the growth cabinets for the years 2011 (a), 2050 (b) and 2100 (c). SE is standard error. (TIF) [file pone.0155131.s001.tif]
